# Fuzzy Entropy-Based Spatial Hotspot Reliability

**DOI:** 10.3390/e23050531

**Published:** 2021-04-26

**Authors:** Ferdinando Di Martino, Salvatore Sessa

**Affiliations:** 1 Dipartimento di Architettura, Università degli Studi di Napoli Federico II, Via Toledo 402, 80134 Napoli, Italy; sessa@unina.it; 2 Centro Interdipartimentale di Ricerca in Urbanistica Alberto Calza Bini, Università degli Studi di Napoli Federico II, Via Toledo 402, 80134 Napoli, Italy

**Keywords:** hotspots, fuzzy clustering, FCM, EFCM, fuzzy entropy, reliability

## Abstract

Cluster techniques are used in hotspot spatial analysis to detect hotspots as areas on the map; an extension of the Fuzzy C-means that the clustering algorithm has been applied to locate hotspots on the map as circular areas; it represents a good trade-off between the accuracy in the detection of the hotspot shape and the computational complexity. However, this method does not measure the reliability of the detected hotspots and therefore does not allow us to evaluate how reliable the identification of a hotspot of a circular area corresponding to the detected cluster is; a measure of the reliability of hotspots is crucial for the decision maker to assess the need for action on the area circumscribed by the hotspots. We propose a method based on the use of De Luca and Termini’s Fuzzy Entropy that uses this extension of the Fuzzy C-means algorithm and measures the reliability of detected hotspots. We test our method in a disease analysis problem in which hotspots corresponding to areas where most oto-laryngo-pharyngeal patients reside, within a geographical area constituted by the province of Naples, Italy, are detected as circular areas. The results show a dependency between the reliability and fluctuation of the values of the degrees of belonging to the hotspots.

## 1. Introduction

Hotspot detection is an emerging spatial analysis feature that allows for the detection of areas in which events representing a certain phenomenon are present with greater insistence (hotspots) and follows their spatial distribution and displacement over time. Cluster techniques are proposed by various researchers to locate hotspots in the study area for many problems. For example, in crime analysis, it is used specifically to locate as hotspots the areas with greater presence and frequency of criminal events in city contexts; in disease analysis, it is used to evaluate the formation and displacement of disease strains over time; in monitoring problems of natural and environmental disasters, such as the monitoring of developments of fires in wooded areas in summer, it is applied to analyze where and with what frequency and intensity natural and malicious phenomena of fires develop.

Cluster algorithms are proposed by some authors to detect hotspots in various spatial analysis problems.

K-means [1] is applied by some authors to detect hotspots in crime analysis [2,3] and fire analysis [4,5]. K-medoids [6] is applied in disease analysis [7]. Fuzzy C-means (for short, FCM) [8,9,10] is applied to detect hotspots in crime analysis [11,12,13,14], road traffic crashes [15], and disease analysis [16]. Kernel density-based algorithms [17] are applied in crime analysis [18], soil pollution [19], and traffic accident analysis [20].

Kernel density algorithms have the advantage of detecting even hotspots of irregular geometric shape, but they are computationally more expensive than K-means and FCM; on the other hand, K-means and FCM detect only cluster centers and are less robust than the presence of noise in the data. Furthermore, in K-means and FCM, the number of clusters must be fixed in advance and validity measures must be used to evaluate what the suitable number of clusters might be.

In [21], a new hotspot detection technique is proposed, based on an extension of FCM, called Extended Fuzzy C-means (for short, EFCM) [22]. EFCM detect cluster as hyper-spheres in the space of the features; the number of clusters must not be set a priori as it is obtained through merging processes of the most similar clusters carried out during each iteration. In [21], the authors show that EFCM can approximate the shape of hotspots on the map and is robust with respect to the presence of noise and outliers. The EFCM hotspot detection method was applied in disease analysis [23,24] and in earthquake disaster analysis [25].

One of the main needs in hotspot detection is to evaluate the reliability of the results by measuring how significant the detected hotspots are. EFCM detects circular hotspots on the map but does not give information about their reliability. This assessment is sometimes interpretative; it is left to the expert who assesses whether the analyzed event persists more frequently in the region where the hotspot was detected. An effective measure of reliability of a hotspot is critical to understanding how accurate the location of the area in which the analyzed phenomenon exists is, in order to monitor it and follow its movements over time. Currently, no hotspot detection method proposed in the literature allows us to measure the reliability of the detected hotspots; a quantitative assessment of the reliability of the detected hotspots is crucial because it would allow the decision maker to evaluate how accurate the geographic location and areal size of a hotspot can be.

In this research we propose a measure of the reliability of the hotspots detected by running the EFCM algorithm, in which the De Luca & Termini fuzzy entropy [26,27] is applied; the reliability of the hotspot is higher if the fuzzy entropy measured for the corresponding cluster is lower. 

Recently, measures of fuzzy entropy of clustering in FCM have been proposed in [28,29]. Following [28], in this work each fuzzy cluster constitutes a fuzzy set in the domain of the data points and a measure of the fuzzy entropy is applied to each fuzzy cluster to evaluate its fuzziness.

If $H\left(A_{i}\right)$ is the fuzziness of the *i*th cluster, normalized in the interval [0,1], we assign a reliability of the correspondent hotspot $A_{i}$ given by:

$$R\left(A_{i}\right)=\;1-H\left(A_{i}\right)$$

The reliability of $A_{i}$ is normalized in the interval [0,1]. It holds 1 when the fuzziness of $C_{i}$ is null (the cluster is a not null crisp set) and 0 when the fuzziness is maximum (all the data points belong to the cluster with a membership degree ½n).

We’ve implemented our method in a GIS-framework in which the EFCM-based hotspot detection algorithm was encapsulated. After executing EFCM, the detected hotspots are shown as circles on the map and the reliability of each hotspot is calculated as in (1). Finally, the hotspot reliability map is constructed.

In next section, the EFCM algorithm and the De Luca & Termini Fuzzy Entropy are introduced. Our framework is described in Section 3. Section 4 show the results of our tests. Finally, considerations are included in Section 5. 

## 2. Preliminaries

### 2.1. The EFCM Algorithm

The EFCM algorithm [22] is a variation of FCM in which the cluster prototypes are hyper-spheres in the space of the features; each cluster is characterized by a vector characterizing its center and its radius.

Let ***X*** = {***x***_1_, …, ***x****_N_*} ⊂ *R^n^* be a set of *N* data points in the n-dimensional space of the features *R^n^* where ***x****_k_ = (x_k_*_1_, …, *x_kn_)*. Let ***V*** = {***v***_1_, …, ***v****_C_*} ⊂ *R^n^* be the set of centers of the *C* clusters. Let ***U*** be the *C* × *N* partition matrix where *u_ik_* is the membership degree of the *k*th data point ***x_k_*** to the *j*th cluster ***v****_j_*. Let ***r*** = {*r*_1_, …, *r_C_*} be the set of radii of the *C* clusters. 

EFCM minimize the following objective function:

(1) $$J\left(U,V,r\right)=\overset{C}{\underset{i=1}{\sum }}\overset{N}{\underset{j=1}{\sum }}u_{ij}^{m}\delta_{ij}^{2}$$

where *m* is the fuzzifier parameter and *δ_ij_*, interpreted as the distance between the *i*th cluster and the *j*th data point, is given by:

(2) $$\delta_{ij}=max\left(0,\;d_{ij}-r_{i}\right)$$

In (2) *d_ij_* is the Euclidean distance between the center of the *i*th cluster and the *j*th pattern and *r_i_* is the radius of the *i*th cluster.

If ***P_i_*** is the covariance matrix of the *i*th cluster:

(3) $$P_{i}=\frac{\sum_{j=1}^{N}u_{ij}^{m}\left(x_{j}-v_{i}\right){\left(x_{j}-v_{i}\right)}^{T}}{\sum_{j=1}^{N}u_{ij}^{m}}$$

then the radius *r**_i_*** of the *i*th cluster is given by:

(4) $$r_{i}=\sqrt{det{\left(P_{i}\right)}^{1/n}}$$

Applying the Lagrange multiplier method to the (1) we obtain the solutions for ***V*** and ***U***:

(5) $$v_{i}=\frac{\sum_{j=1}^{N}u_{ij}^{m}x_{j}}{\sum_{j=1}^{N}u_{ij}^{m}}\;\;\;i=1,\;\ldots ,\;C\;$$

(6) $$u_{ij}=\{\begin{array}{c} \frac{1}{\sum_{k=1}^{C}{\left(\frac{\delta_{ij}}{\delta_{kj}}\right)}^{\frac{2}{\left(m-1\right)}}}\;\;\;if\;\varphi_{j}=0\\ \{\begin{array}{c} 0\;if\;\delta_{ij}>0\\ \frac{1\;}{\;\phi_{j}}\;if\;\delta_{ij}=0 \end{array}\;\;\;if\;\varphi_{j}>0 \end{array}\;\;\;\;i=1,\;\ldots ,\;C,\;\;\;j=1,\;\ldots ,\;N\;$$

where $\phi_{j}$ is the number of cluster whose distance from the *j*th data point $\delta_{ij}\;i=1,\ldots ,\;C$ is equal to 0.

In [22], with the aim to ensure the separation between clusters, the radius of the *i*th cluster calculated at the *t*th iteration, *r_i_^(t)^* is increased by a factor $\frac{\beta^{\left(t-1\right)}}{C^{\left(t-1\right)}}$, where *C*^(*t−*1)^ is the number of clusters detected during the previous iteration and *β*^(*t−*1)^ is the value calculated at the previous iteration of a parameter defined recursively, where $\beta^{\left(0\right)}=1,\;\beta^{\left(\tau \right)}=min\left(C^{\left(\tau -1\right)},\beta^{\left(\tau -1\right)}+1\right)$. 

The optimal number of clusters is found merging at any iteration with the two most similar clusters, under some conditions.

The similarity between two clusters is measured by the following inclusion index:

(7) $$S_{ik}=\frac{\sum_{j=1}^{N}min(u_{ij},u_{kj})}{min\left(\sum_{j=1}^{N}u_{ij},\sum_{j=1}^{N}u_{kj}\right)}\;\;\;\;i=1,\;\ldots ,\;C\;\;\;j=1,\;\ldots ,\;N\;$$

where the similarity cluster matrix **S** is a symmetric matrix. 

Let **S**^(t)^ be the similarity cluster matrix calculated at the *t*th iteration and let i* and k* be the indices of the two most similar clusters; these two clusters are merged if their similarity is greater than an adaptive similarity threshold *α*^(*t*)^ = 1/(*C*^(*t*)^ − 1), and the absolute difference $\left|S_{i^{*}k*}^{\left(t\right)}-S_{i^{*}k*}^{\left(t-1\right)}\right|$ is less than an error η.

When two clusters are merged, the number of clusters is reduced by one unit and the parameter *β* remains unchanged, otherwise, if $S_{i^{*}k*}^{\left(t\right)}>\alpha^{\left(t\right)}$, the parameter *β* is increased, by setting $\beta^{\left(t\right)}=min\left(C^{\left(t-1\right)},\beta^{\left(t-1\right)}+1\right)$. 

If the two most similar clusters are merged:

(8) $$\{\begin{array}{l} u_{i*j}^{\left(t\right)}=u_{i*j}^{\left(t\right)}+u_{k*j}^{\left(t\right)}\;\;\;\forall j\in \left\{1,\ldots ,N\right\}\\ C^{\left(t\right)}=C^{\left(t-1\right)}-1\\ \beta^{\left(t\right)}=\beta^{\left(t-1\right)} \end{array}$$

otherwise:

(9) $$\{\begin{array}{l} if\;S_{i^{*}k*}^{\left(t\right)}>\alpha^{\left(t\right)}\;then\;\beta^{\left(t\right)}=min\left(C^{\left(t-1\right)},\beta^{\left(t-1\right)}+1\right)\;\\ C^{\left(t\right)}=C^{\left(t-1\right)} \end{array}$$

The EFCM algorithm (Algorithm 1) is described in the following pseudocode.
**Algorithm 1: EFCM.**1. Set m, ε, η, the initial number of clusters C^(0)^
2. β ← 1, S* ← 0, S*^prev^ ← 13. Initialize randomly the partition matrix **U** and the centers **v**_i_4. **Repeat**5.   **For** i = 1 to C   //calculate centers and radius of clusters6.     Calculate the center of the *i*th cluster **v**_i_ by (4)7.     Calculate the radius of the *i*th cluster r_i_ by (12)8.     r_i_ ← r_i_ ∙ β/C   //enlarge the radius of the *i*-th cluster9.   **For** i = 1 to C   //calculate new partition matrix10.     **For** j = 1 to N11.       Calculate the membership degree component u_ij_ by (14)12.   **For** i = 1 to C − 1   //Find the two most similar clusters 13.     **For** k = i + 1 to C14.       Calculate S_ik_ by (15)15.       **If** S_ik_ > S* **Then**
16.         S* ← S_ik_17.   **If** |S* − S*^prev^| < η18.     α = 1/(C − 1)19.     **If** S* > α     //merge the two most similar clusters20.       **For** j = i + 1 to N21.         u_ij_ ← u_ij_ + u_kj_22.       **Remove** the *k*th row from **U**23.       C← C − 124.     **Else**25.       β ← min(C, β + 1)26.  **Until**
$\left|U^{\left(t\right)}-U^{\left(t-1\right)}\right|>\varepsilon$
27. **Return** the partition matrix and the volume prototypes of the final C Clusters

### 2.2. De Luca & Termini Fuzzy Entropy and the Measure of Fuzziness

Let F(*X*) = {A: *X* → [0,1]} be the family of fuzzy sets defined on a universe of discourse *X*.

Let *h*: [0,1] → [0,1] be a continuous function called *fuzzy entropy function*. The following restrictions are required for the function h:

1. *h*(1) = 0
2. *h*(u) = *h*(1 −*u*)
3. *h* is monotonically increasing in in [0, ½]
4. *h* is monotonically decreasing in in [½, 1]

The fuzzy entropy function has a minimal value of 0 when u is 0 or 1, and a maximum value when *u* = ½.

De Luca and Termini in [26,27] propose the following fuzzy entropy function:

(10) $$h(u)=\{\begin{array}{ll} 0 & if\;u=0\\ -u\cdot log_{2}(u)-(1-u)\cdot log_{2}(1-u) & if\;0<u<1\\ 0 & if\;u=1 \end{array}$$

which has the maximum value 1 when *u* = ½; it is called Shannon’s function.

If *X* = {$x_{i},x_{2},\ldots ,x_{N}$} is a discrete set, we define the entropy measure of fuzziness of the fuzzy set *A* as:

(11) $$H\left(A\right)=K\overset{N}{\underset{i=1}{\sum }}h\left(A\left(x_{i}\right)\right)$$

where *K* is a multiplicative constant. If *H(A)* = 0, then for each element *x_i_*, *i* = 1, …, *N*, *A(x_i_)* = 0 or *A(x_i_)* = 1 and A coincides with a subset of the set X; if for each element *x_i_*, *i* = 1, …, *N*, *A(x_i_*) = ½ and the fuzziness of *A* is maximum. 

If *A* is a crisp set, its fuzziness is null and *H(A)* = 0. The higher the fuzziness of a fuzzy set, the closer the mean membership degree to the fuzzy set of X’s elements approaches ½.

In [28], a fuzziness measure (12) is used to construct a new validity index, which is applied to evaluate the optimal number of clusters in FCM. If *A_i_* is the *i*th fuzzy cluster where *i* = 1, …, *C* considered as a fuzzy set and u_ij_ is the membership degree of the *j*th data point to the *i*th cluster, the authors use the following fuzzy entropy measure of *A_i_*:

(12) $$H\left(A_{i}\right)=\frac{1}{N}\sum_{j=1}^{N}h\left(u_{ij}\right)\;\;\;i=1,\;\ldots ,\;C$$

where *N* is the number of data points and the De Luca & Termini fuzzy entropy function (11) is used.

## 3. The Proposed Framework

**Figure 1:**
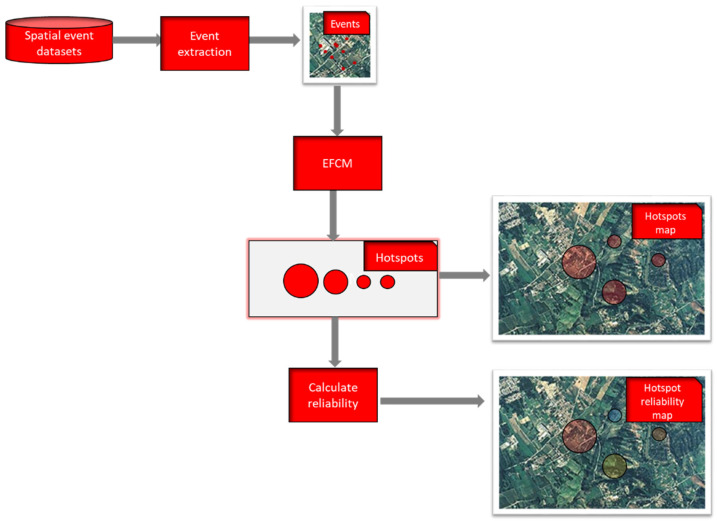
The proposed framework.

We constructed a GIS-based framework in which EFCM is implemented to detect hotspots and the fuzzy entropy measure (13) is calculated to evaluate the reliability of the detected hotspots. The framework is schematized in Figure 1.

The set of spatial events is extracted from the spatial event datasets. Each event is a data point given by its latitude and longitude coordinates. The event extraction functionality builds the set of data points by extracting the event data located in the study area and transforming them into a single system of geographic coordinates. 

The set of spatial events is given by a set of N events ***X*** = {***x***_1_, …, ***x****_N_*} *R^N^*. Each data point is given by a pair ***x****_j_* = (*x_j_*_1_, *x_j_*_2_), *j* = 1, …, *N*; *x_j_*_1_ and *x_j_*_2_ are the latitude and longitude coordinates where the event is located.

EFCM was executed to detect circles C as clusters; each cluster is identified by its center ***v****_j_* = (*v_i_*_1_, *v_i_*_2_), *j* = 1, …, *C* and its radius *r_i_* and the component *u_ij_* of the *C* × *N* partition matrix ***U*** gives the membership degree of the *j*th data point to the *i*th cluster. The cluster prototypes constitute hotspots of circular areas which are shown on the map.

The *Calculate reliability* function calculates the reliability of each hotspot by applying the Formula (13) to assess the fuzziness of the hotspots. The reliability *R*(*A_i_*), *i* = 1, …, *C*, assigned to the *i*th hotspot is given by the Formula (1); it is a value in the range [0,1].

Finally, the reliability thematic map was produced. Below we show the algorithm applied to extract the hotspots assessing their reliabilities (Algorithm 2).
**Algorithm 2: Hotspots reliability evaluation.**1.   Extract the dataset of event **X** = {**x**_1_, …, **x**_N_} ⊂ R^2^2.   Execute EFCM (**X**)3.   **For** i = 1 to C4.     H ← 05.     **For** j = 1 to N6.       H_i_ ← H_i_ + h(u_ij_)//where the Equation (11) is applied for the function h(u)7.     H ← H/N8.     R_i_ ← 1 − H9.   **Return** the centers **v**_i_, the radius r_i_ and the reliability R_i_ i = 1, …, C

In next section we show the obtained results.

## 4. Test Results

We tested our framework on an area of study given by the district of Naples, Italy. The extension of the district is 1171 km^2^. The event dataset was constructed by considering the places of residence of patients who have been diagnosed with oto-laryngo-pharyngeal disease diagnosis in the last four years. These data were collected by entering only non-sensitive information and transmitted by hospitals and medical facilities. An address locator geocoding function was used to geo-refer the data points. 

The event dataset is made from about 4000 data points. The GIS framework was constructed using the tool GIS Esri ArcGis Desktop 10.8; the EFCM algorithm was implemented in the GIS platform using Python libraries.

After executing EFCM, 24 hotspots were detected and plotted as circles on the map.

**Figure 2:**
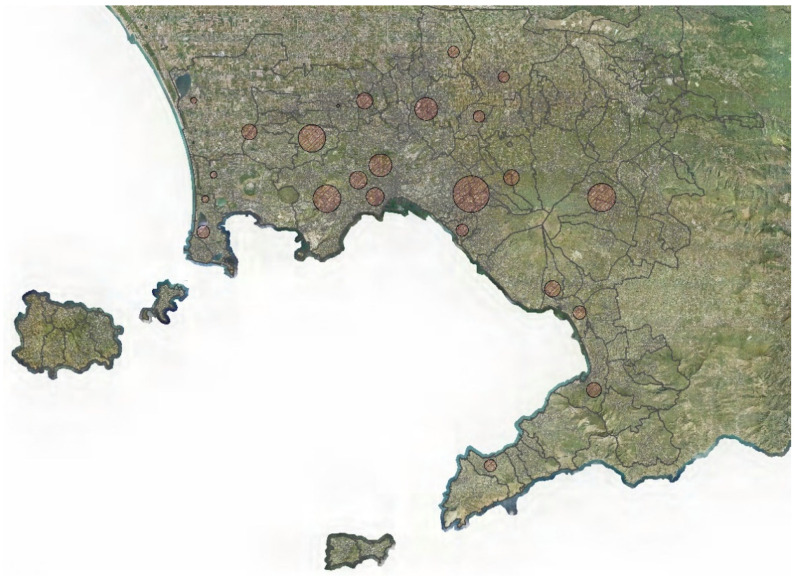
Thematic map with the detected hotspots.

The thematic map with the hotspots is shown in Figure 2.

The detected hotspots have an extension between 0.6 and about 9 km^2^. The area and the reliability of each hotspots are shown in Table 1.

**Figure 3:**
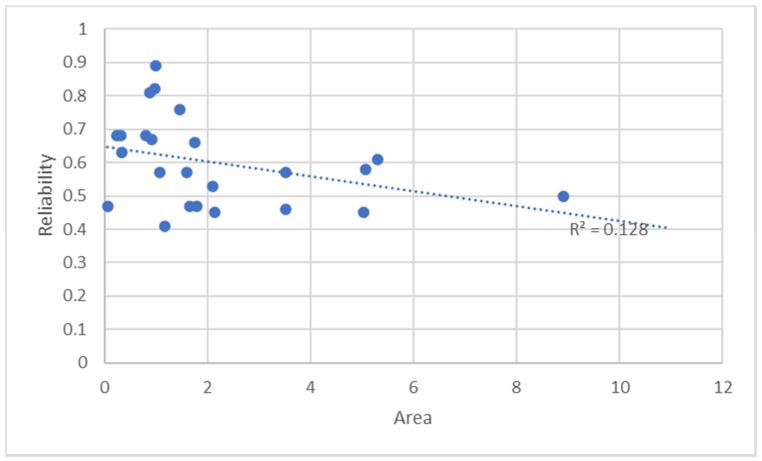
Linear dependency analysis results of the reliability on the area of the hotspots.

Figure 3 shows a plot graph in which the reliability dependency on hotspots area is analyzed.

Figure 3 shows that there is no linear dependency between the area of hotspots and their reliability; the very low value of the coefficient of determination R^2^ (=0.128) means that the smaller and more compact hotspots do not necessarily have greater reliability. 

**Figure 4:**
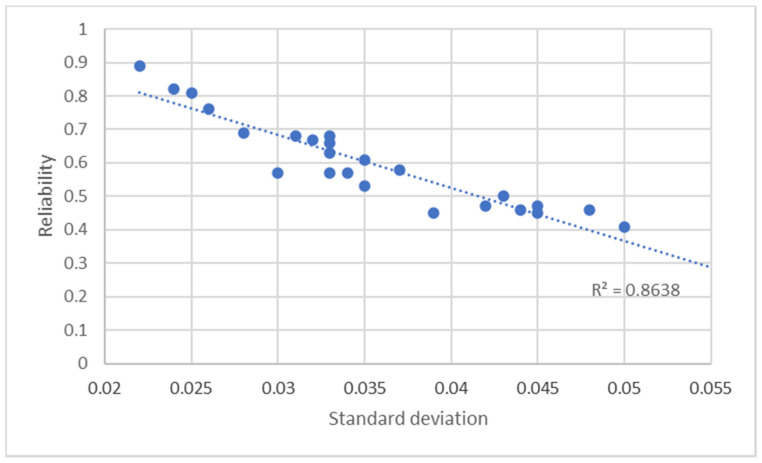
Linear dependency analysis results of the reliability on the standard deviation.

In Figure 4, the graph analyzes the linear dependency of reliability on the standard deviation. The graph shows the presence of a linear relationship between standard deviation and reliability with a mean-high value of the coefficient of determination R^2^ (=0.864): this result means that, on average, the greater the fluctuation of the values of the degrees of belonging of the data points to the hotspot, the greater the fuzzy entropy of the hotspot, therefore the lower its reliability. 

**Figure 5:**
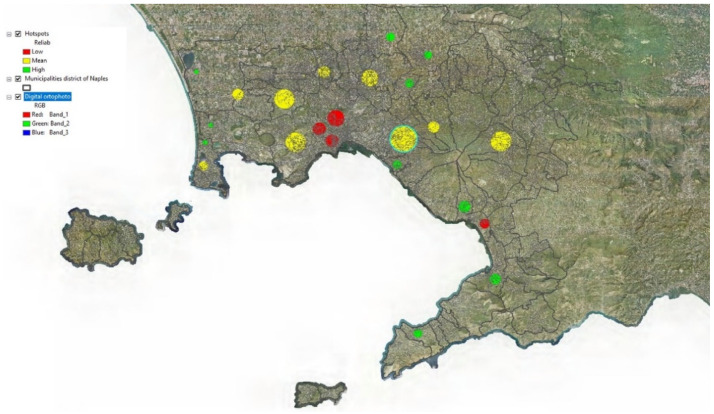
Thematic map with the detected hotspots.

Figure 5 shows a thematic map of the hotspots in which three thematic classes are used, obtained by applying the Jenks Natural Breaks Classification method [30]:

- *Low*, which includes hotspots with reliability less than 0.45
- *Mean*, which includes hotspots with reliability between 0.45 and 0.6
- *High*, which includes hotspots with reliability greater than 0.6

This can provide information on the distribution in the study area of hotspots with different reliabilities. Hotspots with low reliability can be interpreted as hotspots in which there is greater uncertainty regarding their location and extent; on the contrary, hotspots with high reliability are hotspots whose location and extent have been detected with greater certainty. 

The map in Figure 5 shows a concentration of hotspots with low reliability within the municipality of Naples; they are located in an area corresponding to the historic center of the city.

We asked a team of expert doctors who analyzed the dataset of the locations of patients diagnosed with the disease to evaluate how accurate the location and width of each hotspot detected on the map was, assigning one of the three labels: Low, Mean, and High, to each hotspot. In Table 2, the evaluations of the experts are compared with the results obtained in the thematic map in Figure 5. 

The results obtained are correlated with the deductions made by the team of experts. The four hotspots rated with reliability Low, are also evaluated with Low reliability by the pool of experts, who found it more difficult to locate disease strains in the areas including the historic center of the municipality of Naples, due mainly to a high population density, on average homogeneous to the entire area of the historic city center. 

## 5. Final Considerations

To assess the reliability of the hotspots detected in spatial analysis, we propose a framework in which the EFCM hotspot detection algorithm is used to detect the hotspots, and the De Luca and Termini’s fuzzy entropy is applied to measure the reliability of the detected hotspots. 

We tested our framework in a disease analysis problem; the results show that the presence of an approximately linear dependence between the reliability of the detected hotspots and the fluctuation of the membership degrees of the event data points to the corresponding fuzzy clusters. Furthermore, the spatial distribution of the reliability of the detected hotspots corresponds to the assessments made by the pool of experts.

In the future, we intend to adapt and apply our framework on massive event datasets, and to test the proposed method for measuring the reliability of predictions of future locations and displacements of hotspots.

## Figures and Tables

**Table 1:** Area and reliability of the detected hotspots.

ID	Area (km^2^)	Standard dev.	Reliability
01	0.06	0.045	0.47
02	0.24	0.028	0.69
03	0.31	0.031	0.68
04	0.34	0.033	0.63
05	0.81	0.033	0.68
06	0.89	0.025	0.81
07	0.92	0.032	0.67
08	0.97	0.024	0.82
09	1.00	0.022	0.89
10	1.08	0.033	0.57
11	1.18	0.050	0.41
12	1.47	0.026	0.76
13	1.59	0.034	0.57
14	1.66	0.044	0.42
15	1.76	0.033	0.66
16	1.79	0.042	0.43
17	2.10	0.035	0.53
18	2.14	0.039	0.47
19	3.51	0.030	0.57
20	3.52	0.048	0.46
21	5.03	0.045	0.43
22	5.07	0.037	0.58
23	5.30	0.035	0.61
24	8.91	0.043	0.50

**Table 2:** Hotspot reliability Comparison results.

ID	Reliability	Reliability Class	Expert Evaluation
01	0.47	Mean	Mean
02	0.69	High	High
03	0.68	High	High
04	0.63	High	Mean
05	0.68	High	Mean
06	0.81	High	High
07	0.67	High	Mean
08	0.82	High	High
09	0.89	High	High
10	0.57	Mean	Mean
11	0.41	Low	Low
12	0.76	High	High
13	0.57	Mean	High
14	0.43	Low	Low
15	0.66	High	Mean
16	0.42	Low	Low
17	0.53	Mean	Mean
18	0.47	Mean	Mean
19	0.57	Mean	Mean
20	0.46	Mean	Mean
21	0.43	Low	Low
22	0.58	Mean	Mean
23	0.61	Mean	High
24	0.50	Mean	Mean

## Data Availability

This study did not report any data.
